# Optimising secondary prevention in the acute period following a TIA of ischaemic origin

**DOI:** 10.1136/bmjsem-2016-000161

**Published:** 2017-01-06

**Authors:** Neil Heron

**Affiliations:** 1 Department of General Practice and Primary Care, Queen’s University, Belfast, UK; 2 Centre for Public Health Research, Queen’s University, Belfast, UK; 3 Centre of Excellence for Public Health Research, Queen’s University, Belfast, UK

**Keywords:** TIA, minor stroke, secondary cardiovascular prevention, cardiac rehabilitation, SPRITE, ‘The Healthy Brain Manual’.

## Abstract

**Background:**

Transient ischaemic attacks (TIAs) are highly prevalent conditions, with at least 46 000 people per year in the UK having a TIA for the first time. TIAs are a warning that the patient is at risk of further vascular events and the 90-day risk of vascular events following a TIA, excluding events within the first week after diagnosis when the risk is highest, can be as high as 18%. Immediate assessment of patients with TIA, either at accident and emergency, general practice and/or TIA clinics, is therefore required to address secondary prevention and prevent further vascular events.

**Discussion:**

This article addresses the need for optimising secondary prevention in the acute period following a TIA of ischaemic origin to reduce the risk of further vascular events as per recent Cochrane review advice and presents a novel project, Stroke Prevention Rehabilitation Intervention Trial of Exercise (SPRITE), to do this.

**Summary:**

One novel way to tackle vascular risk factors and promote secondary prevention in patients with TIA could be to adapt a cardiac rehabilitation programme for these patients. SPRITE, a feasibility and pilot study (ClinicalTrials.gov Identifier: NCT02712385) funded by the National Institute for Health Research, is attempting to adapt a home-based cardiac rehabilitation programme, ‘The Healthy Brain Rehabilitation Manual’, for use in the acute period following a TIA. The use of cardiac rehabilitation programmes post-TIA requires further research, particularly within the primary care setting.

**What this study adds?**

- Following a transient ischaemic attack (TIA), people are at risk of further cardiovascular events and this is a key time to initiate secondary cardiovascular prevention.
- Previous studies have focused on pharmacological secondary prevention following a TIA.
- However, there is a clear need to initiate non-pharmacological secondary cardiovascular measures, including physical activity promotion, following a TIA.
- One way to promote both pharmacological and non-pharmacological secondary cardiovascular prevention following a TIA is to adapt a home-based cardiac rehabilitation programme, ‘The Healthy Brain Rehabilitation Manual’, for this population following the Medical Research Council guidelines for developing complex health service interventions, which will be done within the Stroke Prevention Rehabilitation Intervention Trial of Exercise study.

## Background

### Definition of transient ischaemic attack

Transient ischaemic attack (TIA) is defined as ‘a transient episode of neurological dysfunction caused by focal brain, spinal cord or retinal ischaemia, without acute infarction’^1^ and is diagnosed by the patients’ history, a neurological examination and/or neuroimaging (typically a CT head scan). Typical symptoms of TIA include the rapid onset of speech disturbance, unilateral weakness or sensory loss, monocular blindness, visual field defect or ataxia.

### Aetiology of TIAs: TOAST classification system

The underlying aetiology of each TIA event can be classified as per the TOAST classification system.^2^ The TOAST classification denotes the underlying cause of the TIA event and this paper focuses on secondary prevention following a TIA due to atherosclerosis or small vessel occlusion, two TOAST subtypes.

### TIA risk factors

TIAs are most commonly caused by the embolic or thrombotic consequences of atherothrombotic disease,^3^ which is similar to the underlying pathological mechanism for cardiovascular disease.^4–6^ In addition to sharing a similar underlying pathological mechanism, cerebrovascular and cardiovascular disease share common underlying risk factors.^5 7^

The modifiable risk factors for all vascular diseases include smoking, excessive alcohol intake, physical inactivity, dietary factors, hypertension, dyslipidaemia, diabetes and obesity^8^ as well as low VO_2max._ 9

Thus, there are several lifestyle modifications that might contribute to a substantial reduction in the risk of vascular events post-TIA and there is evidence that the earlier these interventions can be introduced, the better the outcome.^10^ ^11^

### Pharmacological secondary prevention of stroke

National UK guidelines for the pharmacological treatment of TIA/stroke have been established by the National Institute for Health and Care Excellence (NICE)^12^ and are supplemented by guidelines on tackling individual risk factors. Following the acute diagnosis of TIA, a prophylactic daily dose of 75 mg of aspirin should be initiated and other agents, for example, dipyridamole and clopidogrel, may be added. These agents reduce blood clotting and therefore reduce the chances of a future clot forming within the circulation. Statins should be initiated to lower cholesterol levels^13^ and appropriate antihypertensive medications are used for blood pressure control^14^ as per national management guidelines. However, evidence is growing regarding the contribution of change in modifiable risk factors to reductions in deaths^15^ and there is a need to consider how to promote non-pharmacological measures within secondary prevention.^16^

### Non-pharmacological/ lifestyle risk factors

#### Physical activity

Physical activity promotion and participation must be one of the key goals for modern-day health systems. Indeed, the WHO in 2010^17^ identified physical inactivity as the fourth leading risk factor for global mortality and this equates to 6% of global deaths. Following a TIA, patients should be encouraged to achieve at least the minimum recommended levels of physical activity as established by the chief medical officers and the departments of health.^18^

#### Cardiorespiratory fitness

Related to physical activity is cardiorespiratory fitness, which can be measured by VO_2max_, and is inversely correlated with mortality,^19–22^ the progression of carotid atherosclerosis^23^ and the risk of stroke.^24^ Myers *et al*^25^ found that in male subjects with and without cardiovascular disease, peak exercise capacity after adjustment for age was the strongest predictor of the risk of death and each one metabolic equivalent increase in exercise capacity conferred a 12% improvement in survival.

Exercise, a form of physical activity, can increase VO_2max_ in sedentary persons^26^ and in subacute stroke survivors.^27^

#### Smoking

Smoking is a well-recognised vascular risk factor. The landmark prospective observational study by Doll *et al*^28^ found that British male doctors born between 1900 and 1930 who continued to smoke had a life expectancy 10 years less than that of lifelong non-smokers. Smoking as a vascular risk factor has been continually supported by other studies,^15^ ^29^ ^30^ and patients with TIA should be advised about smoking cessation.^31^

#### Diet

With regards to diet, a recent meta-analysis has shown that dietary fibre is inversely correlated with the risk of stroke,^32^ with fish oils also being protective.^33^ Indeed the ‘Mediterranean diet’ has shown favourable effects on cardiovascular risk factors.^34^ Moreover, hypercholesterolaemia, of which dietary intake may be a source, is a modifiable risk factor for cardiovascular and cerebrovascular diseases.^35^ Cholesterol levels were found to be positively associated with the risk of non-haemorrhagic stroke,^36^ and dyslipidaemia was also a significant risk factor for ischaemic stroke in the INTERSTROKE study.^37^ Patients with TIA should, therefore, be advised accordingly about their dietary habits.

#### Stress

Psychological distress is a well-known risk factor for TIAs. In the observational study by Everson-Rose *et al*,^38^6749 adults free of vascular disease at baseline in the USA, aged 45–84 years old, were followed up for a median of 8.5 years as part of the Multi-Ethnic Study of Atherosclerosis. The authors found that higher levels of stress and depressive symptoms were associated with increased TIA risk, independent of other known vascular risk factors. Moreover, the diagnosis of TIA often leaves survivors with stress, anxiety and depressive symptoms. Indeed, a recent systematic review^39^ has highlighted the prevalence of these often forgotten symptoms following a TIA and/or stroke diagnosis. Patients should therefore be educated about the signs and symptoms to be aware of and signposted appropriately for further management, with the general practitioner often as their first contact.

#### Alcohol

Alcohol excess is a well-known modifiable vascular risk factor, including for TIAs. Gill *et al*^40^ report the ‘J-shaped’ association between alcohol and risk of stroke in a case–control study of approximately 1200 patients, that is, low alcohol consumption may have a protective effect for cerebrovascular events, whereas heavy consumption predisposes to TIAs. Safe alcohol consumption levels should therefore be promoted to patients with TIA to reduce the risk of future vascular events.^41^

## Discussion

### The ‘evidence gap’ from research to practice

Despite the knowledge surrounding vascular risk factors and the recognition that TIAs are often the precursors of disabling strokes, more needs to be done in reducing stroke as the leading cause of adult disability.^42^ Indeed the WHO, as part of their 2013 Global Action Plan For the Prevention and Control of Non-Communicable Diseases, is trying to target a 25% relative risk reduction in overall mortality from cardiovascular diseases, including TIAs.^43^

One novel way to tackle vascular risk factors and promote secondary prevention in patients with TIA could be to adapt a cardiac rehabilitation programme for these patients. Indeed cardiovascular and cere­brovascular diseases share common underlying pathological mechanisms and risk factors. Moreover, cardiac rehabilitation after a myocardial infarction results in a statistically significant reduction in reinfarction (OR 0.53), cardiac mortality (OR 0.64) and all-cause mortality (OR 0.74),^44^ and these findings concur with those of a recent Cochrane review.^45^ Although Heran *et al*^45^ report that the studies included in the review mainly comprise middle-aged men, who are generally at low cardiovascular risk and this should be considered when developing future studies in this area. A Cochrane review^46^ also demonstrated that hospital- and home-based cardiac rehabilitation programmes result in similar health gains, with home-based programmes improving adherence to the programme^47^ and promoting longer-term sustainability of health benefits.^48^

The Stroke Prevention Rehabilitation Intervention Trial of Exercise (SPRITE) is a feasibility and pilot study (ClinicalTrials.gov Identifier: NCT02712385) funded by National Institute for Health Research (NIHR), which is attempting to adapt a home-based cardiac rehabilitation programme for use in the acute period following a TIA. The WHO has defined cardiac rehabilitation as the, ‘sum of activity and interventions required to ensure the best possible physical, mental and social conditions so that patients with chronic or post-acute cardiovascular disease may, by their own efforts, preserve or resume their proper place in society and lead an active life'.^49^

NICE have recommended that the components of cardiac rehabilitation should include exercise, health education and stress management,^50^ helping to tackle the known vascular risk factors as previously documented. Health education would include addressing the known modifiable vascular risk factors as well as advice regarding work, mental health and sexual activity.^50^ These components will all be addressed within our adapted home-based cardiac rehabilitation programme, ‘The Healthy Brain Rehabilitation Manual’. Such research has immediate clinical significance and the potential to change guidelines for the management of TIAs, as well as the potential to reduce morbidity and mortality resulting from TIAs, with clear benefit to patients.

## Summary

One novel way to tackle vascular risk factors and promote secondary prevention in patients with TIA could be to adapt a cardiac rehabilitation programme for these patients. SPRITE, a feasibility and pilot study (ClinicalTrials.gov Identifier: NCT02712385), is attempting to adapt a home-based cardiac rehabilitation programme, ‘The Healthy Brain Rehabilitation Manual’, for use in the acute period following a TIA. The use of cardiac rehabilitation programmes post-TIA requires further research, particularly within the primary care setting.
